# The effectiveness of the 0.19 mg fluocinolone acetonide implant in treating non-infectious posterior uveitis: a real-world experience

**DOI:** 10.1186/s12348-024-00409-x

**Published:** 2024-06-04

**Authors:** Igor Kozak, Avinash Gurbaxani, Maya Pandova

**Affiliations:** 1 Moorfields Eye Hospitals UAE, Abu Dhabi, UAE; 2 Moorfields Eye Hospital Dubai, London, UK; 3 Department of Ophthalmology, New Ahmadi KOC Hospital, Ahmadi, Kuwait; 4 Department of Ophthalmology and Vision Science, University of Arizona at Tucson, Tucson, AZ USA

**Keywords:** Intravitreal 0.19 mg fluocinolone implant, Non-infectious uveitis, Posterior uveitis, Safety, Efficacy, Recurrence

## Abstract

**Background:**

The 0.19 mg fluocinolone acetonide (FAc) implant (ILUVIEN) has been approved for prevention of relapse in recurrent non-infectious uveitis affecting the posterior segment of the eye (NIU-PS). There is little data assessing the long-term efficacy and safety of the FAc implant in this indication. Therefore, we conducted a retrospective interventional case review of 18 eyes from 13 patients with NIU-PS treated with the FAc implant at three ophthalmology departments in the Middle East between 2018 and 2021.

**Main text:**

Baseline patient characteristics, including best-corrected visual acuity (BCVA), central retinal thickness (CRT) and intraocular pressure (IOP), were collected at the time of FAc implant administration and at 1–3 months, 6 months and every six months thereafter. The mean time of follow-up was 29.7 ± 14.6 (mean ± SD) months. Over the follow-up, the BCVA significantly increased from month 1 (*P* = 0.002) until month 36 (*P* = 0.024) and remained improving throughout the follow-up period (*P* = 0.004). The CRT significantly decreased from month 1 (*P* = 0.008) until month 12 (*P* = 0.003) and was persistently lower during the follow-up period (*P* = 0.022). Significant improvements in anterior chamber cells (*P* = 0.004) and vitritis scores (*P* = 0.001) were observed by Month 6. Similarly, at Month 12, significant improvements were noted in both parameters as well (anterior chamber cells: *P* = 0.012; vitritis scores: *P* = 0.004). Mean IOP remained relatively stable throughout (*P* = 0.205) the follow-up.

**Conclusions:**

Our results suggest improvements and long-term maintenance in functional and anatomical outcomes with FAc implant with a manageable safety profile in a real-world clinical setting in patients with NIU-PS.

## Introduction

Uveitis describes a group of disorders characterised by intraocular inflammation and is responsible for significant ocular morbidity [1]. The most sight-threatening forms of uveitis are those that affect the posterior structures of the eye, classified anatomically as intermediate, posterior and panuveitis [2]. If not caused by an infectious agent, these are collectively known as non-infectious posterior segment-involving uveitis (NIU) often requiring systemic or local injection/implant-based therapy. Current first-line treatment algorithms in the management of NIU of the posterior segment involve systemic and local corticosteroids. These agents can be used in conjunction with immunosuppressant therapies [3].

The treatment goals for NIU are to control inflammation in order to limit eye tissue damage, maintain disease control and to preserve or improve vision. Cumulative damage from repeated inflammatory episodes affecting the posterior segment has been associated with significant visual morbidity [4]. The ideal treatment is a targeted one which limits systemic exposure, which is long lasting and reduces the dosing frequency, and which is easily administered. Intravitreal drug delivery systems fulfil many of these criteria and may help clinicians meet these goals [5, 6]. To date, a number of corticosteroid-based intravitreal implants have been reported to successfully treat non-infectious uveitis and stabilize disease activity. These include dexamethasone (OZURDEX®) and fluocinolone acetonide (ILUVIEN®, RETISERT®, YUTIQ®) implants [7–9].

The 0.19 mg fluocinolone acetonide (FAc) implant (ILUVIEN®; Alimera Sciences Inc., Georgia, USA) is approved for prevention of relapse in recurrent non-infectious uveitis affecting the posterior segment of the eye [10]. It has the longest release time of all available implants thus making it a good candidate for chronic conditions such as posterior NIU affecting the posterior segment of the eye. With emerging use of FAc implant in this indication, however, the experience and long-term follow-up data are still limited, and many questions remain such as the types of conditions that are treated and the outcomes in patients with bilateral conditions. To addresses these gaps, we present a real-world interventional case series study from the Middle East region (following UK NICE guidelines) with a focus on the outcomes in a mean time of follow-up of 29.7 ± 14.6 months of therapy.

## Patients and methods

### Patient examination, diagnosis and treatment history

This was a retrospective clinical audit of eyes with non-infectious uveitis affecting the posterior segment of the eye that had been treated with a single FAc intravitreal implant injection, between December 16, 2018, and February 10, 2021, at three ophthalmology departments in the Middle East—Moorfields Eye Hospital Abu Dhabi, Moorfields Eye Hospital Dubai and the Department of Ophthalmology, Ahmadi Hospital, KOC, Kuwait. The research protocol was approved by Moorfields Eye Hospital Abu Dhabi Research and Ethics Committee (REC/2021/P24). Patient demographic data and history of previous treatments were recorded prior to FAc therapy. All patients underwent a complete ophthalmic evaluation prior to commencing FAc therapy.

The case review involved a total of 18 eyes with posterior non-infectious uveitis with a mean follow-up of 29.7 ± 14.6 months (range, 3 to 54 months).

Clinical assessments included best-corrected visual acuity (BCVA; using logMAR scale and converted to ETDRS letters), intraocular pressure (IOP) using Goldmann applanation tonometer, slit-lamp and dilated fundus exam, measurements of central retinal thickness (CRT) using spectral-domain optical coherence tomography (SD-OCT) scans (Heidelberg Spectralis, Heidelberg, Germany) and fundus photography (Optos®, Dunfermline, Scotland). The uveitis activity was assessed by scoring anterior cell counts using a 1 × 1 mm slit-lamp light beam per The Standardisation of Uveitis Nomenclature (SUN) Working Group classification [11]. The SUN classification score was also applied for scoring vitreous haze using indirect ophthalmoscopy. Safety was evaluated by observation of any adverse events during treatment or follow-up period.

### Statistical analysis

Descriptive analyses were performed using the Microsoft Excel (Microsoft Corporation, Washington, USA). Numeric values are reported as mean ± SD (standard deviation), unless stated otherwise, with the number of patients reported in parenthesis. Statistical analysis was conducting using Wilcoxon signed ranks test and test of marginal homogeneity for categorical variables. A *P*-value less than 0.05 was taken as being statistically significant.

## Results

### Baseline characteristics and prior therapies

Out of the 18 eyes studied, 9 eyes had a follow-up duration exceeding 36 months, while the remaining eyes were followed up for durations ranging between 3 and 54 months (mean, 29.7 ± 14.6 months) in total 13 patients. Patient demographics and baseline characteristics are shown in Table 1. The patients’ mean age was 50.3 (range, 29 to 84), six were female and the majority of eyes were pseudophakic (n = 14, 77.8%). One patient had proliferative diabetic retinopathy, one had glaucoma and was being managed with IOP-lowering drops at baseline and a vitrectomy had been performed in three eyes prior to FAc implant therapy.

**Table 1:** Baseline patient characteristics

Paremeter	Value (*n* = 18 eyes from 13 patients)
Gender (female)	6
Age, mean ± SD (range), years	50.3 ± 17.4 (29 to 84)
Lens status, % eyes (*n*)	77.8% (14) pseudophakic and 22.2% (4) phakic
Prior vitrectomy, % eyes (*n*)	17% (3)
Diagnoses, *n*	*N* = 4 chronic uveitis (*N* = 2 with known tuberculosis); *N* = 3 panuveitis; *N* = 5 retinal vasculitis/vitritis; *N* = 2 TB uveitis; *N* = 2 MFC
Prior intravitreal therapy, % eyes (*n*)	39% (7) eyes had been treated with a dexamethasone implant. One of those eyes had also been treated with anti-VEGF
Prior systemic therapy, % eyes (*n*)	0.0% (0)
Baseline BCVA, mean ± SD (range), ETDRS letter score	58.4 ± 18 (26.2 to 80.0)
Baseline CRT, mean ± SD (range), µm	407.3 ± 148.6 (231 to 732)
Baseline IOP, mean ± SD (range), mmHg	12.4 ± 5.1 (5 to 23)
Baseline AC scores	*N* = 4 with a score of ≥ 2
	*N* = 4 with a score ≥ 1
	*N* = 9 with a score of 0
Baseline vitritis scores	*N* = 2 with a score of ≥ 3
	*N* = 6 with a score of ≥ 2
	*N* = 4 with a score ≥ 1
	*N* = 1 with a score ≥ 1/2
	*N* = 4 with a score of 0

*CME* cystoid macular edema, *MFC* multifocal choroiditis, *TB* tuberculosis, *VEGF* vascular endothelial growth factor, *FAc* fluocinolone acetonide, *BCVA* best corrected visual acuity, *SD* standard deviation, *ETDRS* Early Treatment Diabetic Retinopathy study, *CRT* central retinal thickness, *IOP* intraocular pressure, *AC* anterior chamber

A dexamethasone implant had been given to seven eyes (3.0 ± 2.2; range, 1.0 to 7.0 injections) with one of these eyes also receiving anti-VEGF injections (four aflibercept and nine ranibizumab) and another had received systemic therapy (mycophenolate) which had been stopped the year prior to administration of a fluocinolone implant. The indications for implantation are detailed in Table 1. It should be noted that four eyes in the study presented with TB-related uveitis. However, it is important to emphasize that the FAc implant administrated was primarily directed towards eyes with quiescent TB-related uveitis, where the underlying pathophysiology was not primarily infection-related. Prior to FAc implant therapy, twelve of the eighteen eyes had a vitritis score equal ≥ 1 and in eight of the eighteen eyes anterior cell counts were ≥ 1 (Table 1).

### Vision, retinal and intraocular pressures changes following therapy

**Fig. 1:**
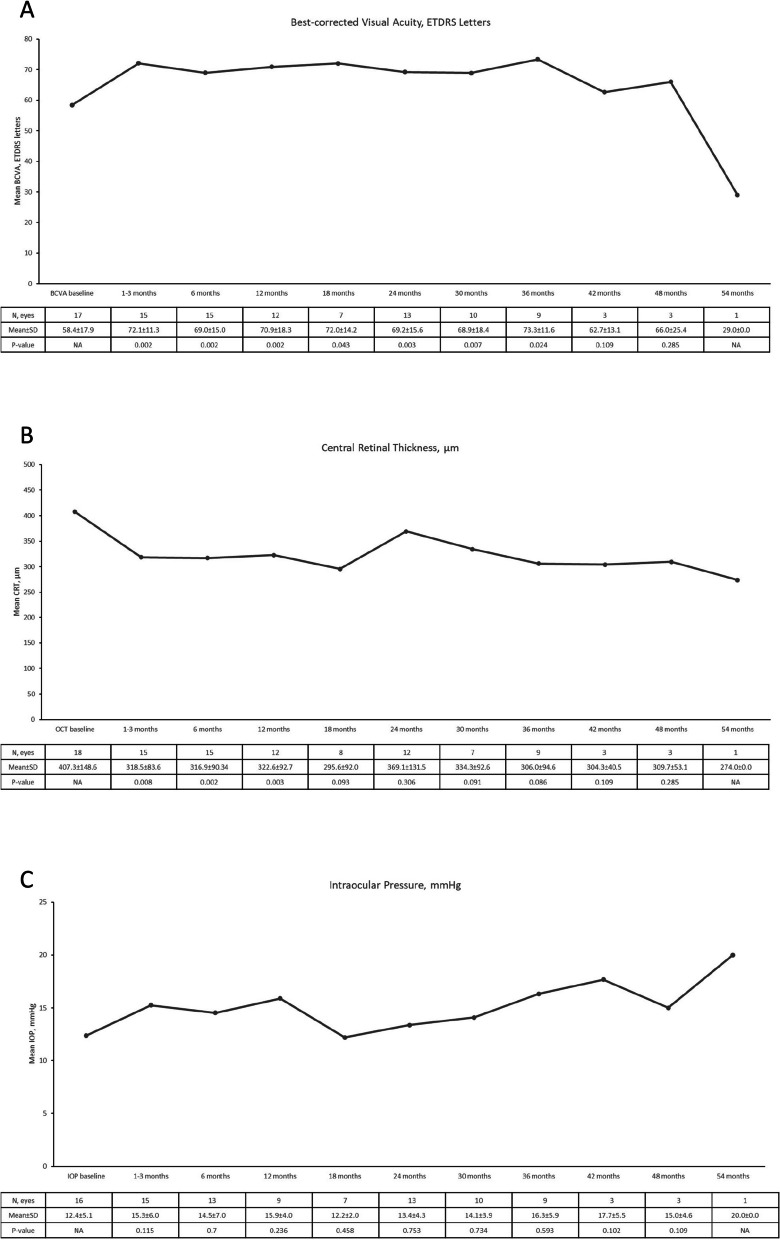
Central retinal thickness, visual acuity and intraocular pressure outcomes. Figure 1a. Visual acuity outcomes. Figure 1b. Central retinal thickness (CRT) outcomes. Figure 1c. Intraocular pressure (IOP) outcomes

Following injection of the fluocinolone implant there were marked improvements in BCVA (Fig. 1a) and CRT (Fig. 1b) with changes seen at the first visit (1 to 3 months after therapy commenced) (Fig. 1). These changes were sustained throughout the follow-up with significant changes in BCVA from 58.4 ± 17.9 ETDRS letters to 67.8 ± 17.9 at ETDRS letters (*P* = 0.004) and CRT of 407.3 ± 148.6 µm to 325.8 ± 133.4 µm (*P* = 0.022) from baseline to last observation, respectively. Mean intraocular pressure was stable throughout (Fig. 1c) with a mean IOP varying from 12.4 ± 5.1 mmHg to 15.3 ± 4.6 mmHg from baseline to last observation (*P* = 0.205). One patient had glaucoma at baseline and was being managed with Xalatan, Cosopt and Alphagan topical therapy. Two other patients received IOP-lowering drops following FAc implant. One patient also received topical anti-inflammatory drops for one month (Nepavenac).

### Anterior cell and vitritis scores following therapy

**Fig. 2:**
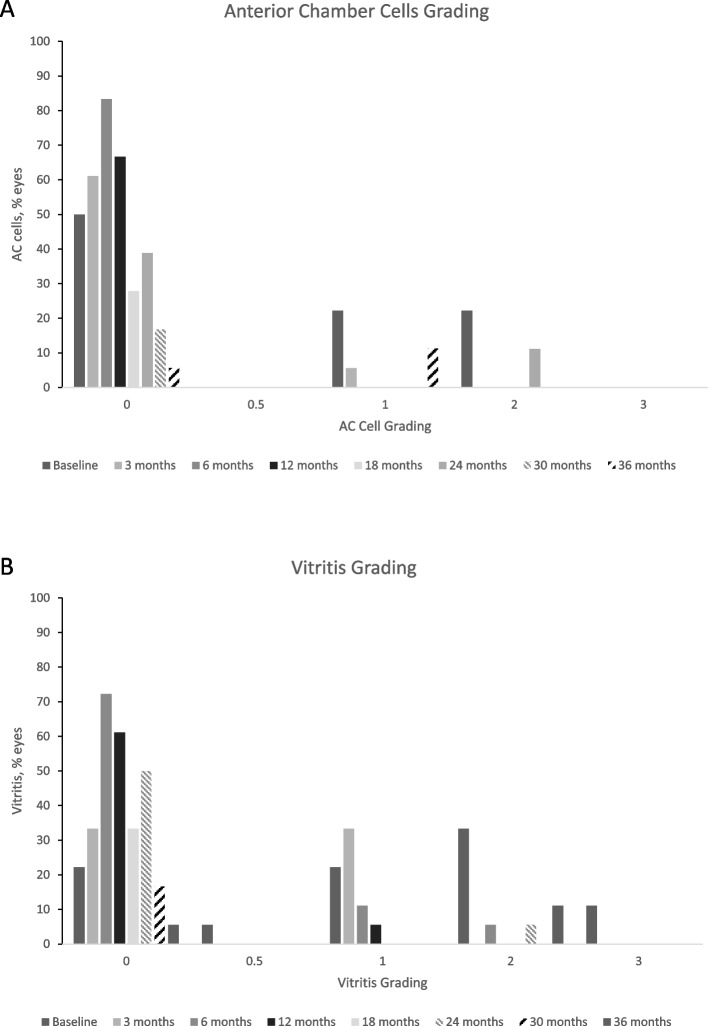
Anterior chamber cells and vitritis gradings over the study period. Figure 2a. Proportion of patient eyes by anterior chamber cells grading. Note: number of patient eyes: baseline, *n* = 17; month 3, *n* = 12; month 6, *n* = 15; month 12, *n* = 12; month 18, *n* = 5; month 24, *n* = 9; month 30, *n* = 3 and month 36, *n* = 3. Figure 2b. Proportion of patient eyes by vitritis grading. Note: number of patient eyes: baseline, *n* = 17; month 3, *n* = 12; month 6, *n* = 16; month 12, *n* = 12; month 18, *n* = 6; month 24, *n* = 10; month 30, *n* = 3 and month 36, *n* = 3

Figure 2 shows the mean anterior cell (AC) and vitritis scores at baseline and following therapy. Nine eyes had a mean AC count ≥ 1 at baseline. By Month 6, fifteen eyes (83.3%) had a score equal to zero and this was sustained to Month 12 (Fig. 2a). Indeed, the proportion of eyes with an AC cell score ≥ 1 decreased from 9 eyes at baseline to 0 eyes by Month 12 (*P* = 0.012). These improvements were also observed in mean vitritis scores. Twelve eyes had a vitritis score ≥ 1 at baseline and by Month 6 thirteen eyes (72.2%) had a score equal to zero and by Month 12 eleven eyes (61.1%) had a zero score. Like AC cells, the proportion of eyes with a vitritis cell score ≥ 1 decreased from 12 eyes at baseline to one eye by Month 12 (*P* = 0.004, Test of Marginal Homogeneity).

### Other findings

Additional therapies were required in four eyes (mean 12.4 ± 4.2; range, 7.0 to 16.0 anti-VEGF injections). Three eyes were reinjected with FAc implant during the follow-up period (mean time for re-injection was 29.5 ± 0.7 months). Two patient eyes underwent pars plana vitrectomy due to vitreous opacities at 30 months of follow-up. Two patient eyes underwent cataract surgery five months after fluocinolone acetonide implant was administered. In these eyes, cystoid macular edema had worsened. This effect was also observed in the third phakic eye, which did not undergo cataract surgery during this period. Furthermore, one patient eye had a recurrence of uveitis after 8 months, three eyes developed epiretinal membrane and one developed posterior capsule opacification.

### Bilateral patient case

**Fig. 3:**
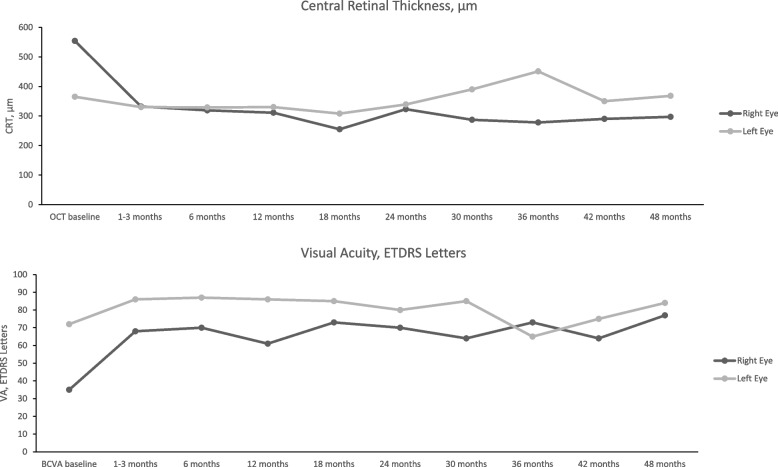
Bilateral case from one patient. Anatomical and functional performance during follow-up

**Fig. 4:**
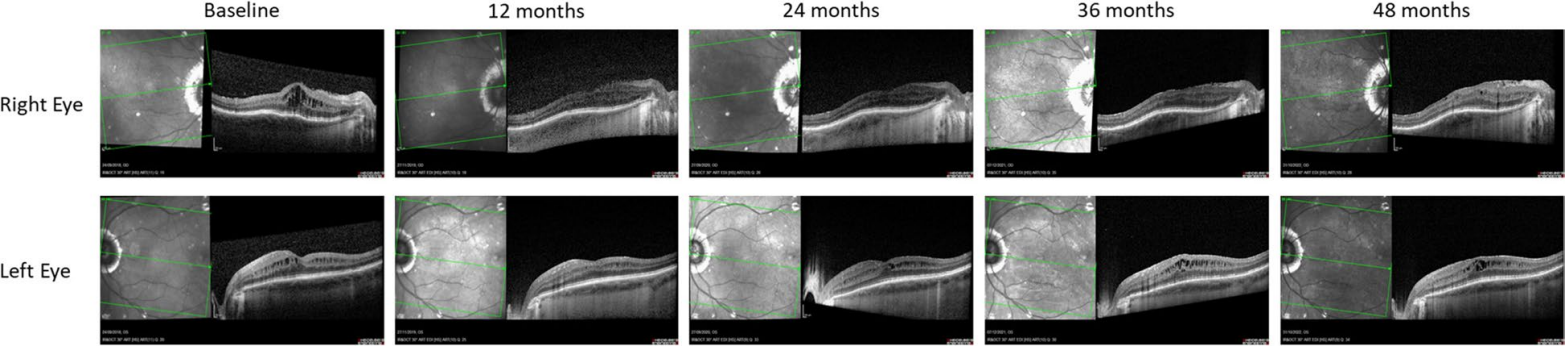
Bilateral case from one patient. Structural performance by spectral-domain optical coherence tomography follow-up

Figures 3 and 4 shows the anatomical and functional performance from one female patient. She was aged 38 years of age and both eyes were pseudophakic at baseline. The patient was diagnosed with bilateral panuveitis. Her previous treatment included 7 bilateral intravitreal dexamethasone implants each leading to a duration of uveitis quiescence of less than 3 months. The patient was treated bilaterally with FAc implants in September 2018. Following treatment, the right eye developed posterior capsule opacification which was treated with YAG laser. The left eye developed an epiretinal membrane.

In terms of structure and function, CRT remained between 330 and 332 µm from the first follow-up time point (i.e., months 1 to 3) through to Month 24. BCVA also improved in both eyes with baseline values improving from 35 and 72 letters at baseline to 68 and 86 letters by the first visit and this was maintained to Month 24 (70 and 80 letters respectively in right and left eyes). It was notable that anterior cell counts were zero by Month 3 and vitritis scores were zero by Month 6. Throughout the study period intraocular pressure remained below 21 mm Hg in both eyes and no IOP-lowering drops were required. Supplemental therapy was required in both eyes (*N* = 6 injections in the right eye and *N* = 7 in the left eye) following therapy with a second FAc implant in both eyes at month 18. During the subsequent 30 months of follow-up, both CRT and BCVA remained stable with no significant increases in IOP. The two eyes underwent pars plana vitrectomy 1 year following the second FAc implant due to the presence of vitreous opacities and both eyes required supplemental therapy (*N* = 1 injection in the right eye and *N* = 2 in the left eye) to manage macular edema.

## Discussion

This study demonstrates that the long-term FAc intravitreal implant is effective for the treatment of NIU in a Middle East population. It has shown to control uveitis relapses through reduced inflammation in association with improved visual acuity and structural parameters of uveitis.

Sustained-release corticosteroid implants have emerged to bridge the problems related to intravitreal injections. These implants reduce the number of injections required and therefore minimize the potential adverse effects of multiple injections. They also help to increase patient comfort and adherence to the treatment by being able to deliver a low dose of the drug whilst helping to reduce drug-related adverse events [12].

In comparison to the 0.59 mg fluocinolone acetonide intravitreal implant (Retisert; Bausch + Lomb, Bridgewater, NJ, USA), which requires scleral anchoring whereas the FAc intravitreal implant is administered as an intravitreal injection and by doing so a lower dose of 0.2 µg/day of fluocinolone acetonide is administered to the patient. Compared with the 0.59 mg implant, the 0.19 mg intravitreal implant is associated with a lower risk of raised IOP, and this helps to reduce the need for medication or surgical intervention to control pressure and/or remove cataract formations [9].

Here we demonstrate that the safety profile over a mean follow-up of 29.7 ± 14.6 months shows no concern for use of this treatment in prevention of relapse in NIU. We did not identify an increased risk for IOP developing in the whole group. One eye, however, developed glaucoma which was successfully controlled with topical therapy while uveitis was controlled, and visual acuity improvement maintained. There was progression of cataract in phakic eyes, and two out of three underwent cataract surgery with improvements in both BCVA and CRT by 36 months of follow-up. Two eyes developed visually non-significant mild epiretinal membrane, one of whom had trauma related uveitis. This observation mirrors safety data from the original trials [13, 14]. Procedure related events such as conjunctival hyperemia and temporary ocular surface discomfort were not raised as a concern in the patients studied, which may be because these patients are used to receiving local anti-inflammatory therapies.

The efficacy data are encouraging with significant improvement in signs of inflammation as well as measures of both structure (i.e., CRT) and function (BCVA). These improvements were observed from Month 3 and were maintained in all studied eyes. The disease profile of our patients is similar to those previously reported in other studies from other regions [15, 16]. Concerns have arisen that the lower dose of fluocinolone acetonide in the FAc implant (0.2 µg/day) might lead to a slower onset of action and weaker therapeutic effects in terms of inflammatory control. As shown here, however, this was not the case with the onset of effects documented at Month 3 with clinical improvements observed even earlier after therapy commenced (i.e., within the 1 to 3 months window after therapy commenced). Furthermore, the control of inflammation seems to also be comparable to current publications and even reports where higher intravitreal corticosteroid doses have been administered [17, 18].

Our findings suggest a notable efficacy in the management of NIU in terms of preventing uveitis relapses during the follow-up period. At Month 6, our case series showed that uveitis was inactive in the vitreous with 72.2% of eyes (*n* = 13 of 18) having a vitritis score less than one and 83.3% of eyes (*n* = 15 of 18) having an AC cell count less than one. Hence, the FAc implant could control intraocular inflammation and maintaining quiescence during the mean follow-up in the majority of our patients. In diabetic patients, ocassional anti-angiogenic injections were used as a rescue therapy for macular edema.

With regard to diagnoses, it is worth noting that the type of uveitis was not homogenous, and improvements were observed across all uveitis indications, which included chronic uveitis (with known tuberculosis), panuveitis, retinal vasculitis/vitritis, tuberculosis uveitis with controlled and inactive infection on four anti-tuberculous medicines, pseudophakic CME/panuveitis and multifocal choroiditis.

Limitations of this report are a lack of control group with no randomization. However, this is meant to be a clinical audit with the focus being on the long-term safety and initial effectiveness in a Middle East population. While the index cohort is relatively small it is comparable to others recently reporting on use of FAc intravitreal implant in NIU and presents a number of patients with a follow-up of > 36 months [16].

In conclusion, we report initial real-world experience with the long-term FAc intravitreal implant. Our data show it was effective for the treatment of NIU in a Middle East population and worked to control uveitis relapses through reduced inflammation and this was associated with improved visual acuity.

## Data Availability

The datasets used and/or analysed during the current study are available from the corresponding author on reasonable request.

## Abbreviations

AC: Anterior chamber cells
BCVA: Best corrected visual acuity
CME: Cystoid macular edema
CRT: Central retinal thickness
ETDRS: Early Treatment of Diabetic Retinopathy Study
FAc: Fluocinolone acetonide
IOP: Intraocular pressure
MFC: Multifocal choroiditis
NIU-PS: Non-infectious uveitis affecting the posterior segment of the eye
OCT: Optical coherence tomography
SUN: The Standardization of Uveitis Nomenclature
SD: Standard deviation
TB: Tuberculosis
VEGF: Vascular endothelial growth factor
